# *De Novo* Whole-Genome Sequence and Genome Annotation of *Lichtheimia ramosa*

**DOI:** 10.1128/genomeA.00888-14

**Published:** 2014-09-11

**Authors:** Jörg Linde, Volker Schwartze, Ulrike Binder, Cornelia Lass-Flörl, Kerstin Voigt, Fabian Horn

**Affiliations:** a Systems Biology/Bioinformatics, Hans-Knöll-Institut, Jena, Germany; b Jena Microbial Resource Collection, Hans-Knöll-Institut, Jena, Germany; c Division of Hygiene and Medical Microbiology, Innsbruck Medical University, Innsbruck, Austria

## Abstract

We report the annotated draft genome sequence of *Lichtheimia ramosa* (JMRC FSU:6197). It has been reported to be a causative organism of mucormycosis, a rare but rapidly progressive infection in immunocompromised humans. The functionally annotated genomic sequence consists of 74 scaffolds with a total number of 11,510 genes.

## GENOME ANNOUNCEMENT

*Lichtheimia ramosa* (formerly *Absidia idahoensis* var. *thermophila, L. hongkongensis*) belongs to the order of *Mucorales* (1). Besides *L. ramosa, L. ornata* and *L. corymbifera* are clinically relevant (1, 2, 3, 4). The virulence potential of this fungus is connected with thermotolerance (5), because other clinically nonrelevant *Lichtheimia* species possess a lower thermotolerance and stop growth at 42°C (1, 6). So far, missing genomic data has hindered the exploration of further virulence factors (7, 8). Here we present the full genome sequence of *L. ramosa*, whereas the mitogenome was announced recently (9).

DNA was obtained from mycelia cultured in liquid supplemented minimal medium (SUP medium) under shaken conditions for 3 days at 37°C (10). One library was prepared for 8-kb Roche/454PE GS FLX+ Titanium sequencing and a second library for Illumina HiSeq 2000 100-bp PE sequencing. Genome sequencing and assembly was generated by LGC Genomics (Berlin) using a hybrid approach. Illumina contigs, assembled by Velvet (11), and 454 scaffolds, assembled by Newbler 2.6 (454 Life Sciences), were merged using Minimus2 (12). The resulting scaffolds were finalized using SOAP GapCloser (13) and SEQuel (14). RNA-Seq data were obtained from a pooled sample cultured under five different conditions. Transcriptome sequencing was performed using Roche/454 GS FLX+ Titanium, and contigs were assembled using Newbler.

For gene prediction, the pipeline presented by Haas et al. (15) was customized, and tools incorporating *ab initio* models, transcriptome data, and protein alignments were applied. The parameter sets were trained using gene models that were predicted by TransDecoder (16) from aligned species-specific transcripts. All gene predictions were combined using EVidenceModeler. Untranscribed regions were added using PASA (17).

For *ab initio* gene prediction, GeneMark-ES (18), Augustus (19), SNAP (20), and Glimmer (21) were applied. Transcriptome data were incorporated into Augustus, FGENESH (22), and PASA. Protein alignments were obtained by mapping proteins from *L. hyalospora* (JGI), *Rhizopus delemar* (BROAD), *Rhizopus microsporus* var. *microsporus* (JGI), *Mucor circinelloides* (JGI), and *Phycomyces blakesleeanus* (JGI) using Exonerate (23) and Scipio (24).

Genes were functionally annotated using Blast2GO (25) and InterproScan (26), including the TMHMM (27) option. Gene descriptions were obtained by blasting the predicted protein sequences against the fungal UniProt Knowledgebase (28). Secondary metabolite gene clusters were predicted using SMURF (29).

454 DNA sequencing resulted in 1,345,023 reads (760 Mbp; estimated genome coverage, 24.3-fold). Illumina DNA sequencing resulted in 426,388,592 raw reads, where 45,982,894 reads passed stringent quality filters (4.10 Gbp; estimated genome coverage, 130-fold) and have been used to create the final assembly. The assembly consists of 74 scaffolds and 30.71 Mbp (*N*_50_, 1.22 Mbp; *N*_90_, 338kbp). The G+C content of the assembly is 41.2%. RNA sequencing and transcriptome assembly led to 12,134 transcripts (11.19 Mbp; estimated transcriptome coverage, 0.5-fold). The final gene prediction consists of 11,510 genes and 11,546 transcripts, and 452 (98.7%) eukaryotic core proteins were identified using CEGMA (30). The coding density of the genome is 52%. Functional names were assigned to 980 transcripts, gene ontology categories to 6,899 transcripts, and protein domains to 9,664 translated transcripts; 2,645 transcripts were predicted to contain transmembrane domains, and 38 transcripts have been assigned to three secondary metabolite gene clusters.

### Nucleotide sequence accession numbers.

This whole-genome shotgun project has been deposited in DDBJ/ENA/GenBank under the accession numbers LK023313 to LK023386. The version described in this paper is the first version. Genome data and additional information are also available at the HKI (Hans-Knöll-Institute) Genome Resource (http://www.genome-resource.de).
